# Therapeutic effects of CD133 + Exosomes on liver function after stroke in type 2 diabetic mice

**DOI:** 10.3389/fnins.2023.1061485

**Published:** 2023-03-09

**Authors:** Poornima Venkat, Huanjia Gao, Elizabeth L. Findeis, Zhili Chen, Alex Zacharek, Julie Landschoot-Ward, Brianna Powell, Mei Lu, Zhongwu Liu, Zhenggang Zhang, Michael Chopp

**Affiliations:** ^1^Department of Neurology, Henry Ford Hospital, Detroit, MI, United States; ^2^Department of Public Health Sciences, Henry Ford Hospital, Detroit, MI, United States; ^3^Department of Physics, Oakland University, Rochester, MI, United States

**Keywords:** liver function, exosomes, stroke, type 2 diabetes mellitus, brain-liver axis, CD133 +

## Abstract

**Background and purpose:**

Non-alcoholic fatty liver disease (NAFLD) is known to adversely affect stroke recovery. However, few studies investigate how stroke elicits liver dysfunction, particularly, how stroke in type 2 diabetes mellitus (T2DM) exacerbates progression of NAFLD. In this study, we test whether exosomes harvested from human umbilical cord blood (HUCBC) derived CD133 + cells (CD133 + Exo) improves neuro-cognitive outcome as well as reduces liver dysfunction in T2DM female mice.

**Methods:**

Female, adult non-DM and T2DM mice subjected to stroke presence or absence were considered. T2DM-stroke mice were randomly assigned to receive PBS or Exosome treatment group. CD133 + Exo (20 μg/200 μl PBS, i.v.) was administered once at 3 days after stroke. Evaluation of neurological (mNSS, adhesive removal test) and cognitive function [novel object recognition (NOR) test, odor test] was performed. Mice were sacrificed at 28 days after stroke and brain, liver, and serum were harvested.

**Results:**

Stroke induces severe and significant short-term and long-term neurological and cognitive deficits which were worse in T2DM mice compared to non-DM mice. CD133 + Exo treatment of T2DM-stroke mice significantly improved neurological function and cognitive outcome indicated by improved discrimination index in the NOR and odor tests compared to control T2DM-stroke mice. CD133 + Exo treatment of T2DM stroke significantly increased vascular and white matter/axon remodeling in the ischemic brain compared to T2DM-stroke mice. However, there were no differences in the lesion volume between non-DM stroke, T2DM-stroke and CD133 + Exo treated T2DM-stroke mice. In T2DM mice, stroke induced earlier and higher TLR4, NLRP3, and cytokine expression (SAA, IL1β, IL6, TNFα) in the liver compared to heart and kidney, as measured by Western blot. T2DM-stroke mice exhibited worse NAFLD progression with increased liver steatosis, hepatocellular ballooning, fibrosis, serum ALT activity, and higher NAFLD Activity Score compared to T2DM mice and non-DM-stroke mice, while CD133 + Exo treatment significantly attenuated the progression of NAFLD in T2DM stroke mice.

**Conclusion:**

Treatment of female T2DM-stroke mice with CD133 + Exo significantly reduces the progression of NAFLD/NASH and improves neurological and cognitive function compared to control T2DM-stroke mice.

## Introduction

Diabetes mellitus (DM) leads to a 3–4 fold higher risk of ischemic stroke (Mast et al., 1995; Goldstein et al., 2006; Adams et al., 2007). Ischemic stroke patients with DM exhibit a distinct risk-factor and etiologic profile and a worse outcome than non-DM patients (Scott et al., 1999; Capes et al., 2001; Putaala et al., 2011). Type 2 DM (T2DM) constitutes ∼90% of diabetic patients and is associated with non-alcoholic fatty liver disease (NAFLD) (Patel et al., 2011). Non-alcoholic steatohepatitis (NASH) is a form of NAFLD in which steatosis in the liver is accompanied by inflammation and cell damage. NAFLD is a chronic liver disease that is present in 50–60% of patients with T2DM, and the two conditions often act synergistically to aggravate the severity of outcomes from ischemic stroke (Megherbi et al., 2003; Putaala et al., 2011; Seo et al., 2016; Li et al., 2018; Weinstein A. et al., 2018; Chatzopoulos et al., 2021). Given the global prevalence of NAFLD, and the increased post-stroke morbidity and mortality in the expanding diabetic population, there is a compelling need to elucidate the brain-liver interaction after stroke in the setting of T2DM and to develop therapeutic approaches specifically designed, not only to reduce stroke induced direct neurological and cognitive deficits, but also to reduce liver damage driven by the interaction of brain and liver.

Exosome based therapeutics is emerging for neurological diseases, cancers, and autoimmune diseases (Peterson et al., 2007; Cheng et al., 2011; Ding et al., 2011; Zhu et al., 2011; Minnerup et al., 2012; Corral-Fernández et al., 2013; Wei et al., 2013). Exosomes are extracellular vesicles naturally released by all cells and they transport nucleic acids, proteins, lipids, and metabolites thereby facilitating intercellular communication (El-Andaloussi et al., 2012; Yáñez-Mó et al., 2015; Kalluri and LeBleu, 2020). Systemic delivery of exosomes that are derived from mesenchymal stromal cells or endothelial cells, primarily accumulate in liver and spleen, and can pass the blood brain barrier without any apparent adverse effects (Xin et al., 2013; Lai et al., 2014; Yang et al., 2015; Venkat et al., 2019; Wang et al., 2020; Choi et al., 2021). CD133 is a marker for hematopoietic stem and progenitor cells, and CD133 + /KDR + identifies endothelial progenitor cells (EPC) (Friedrich et al., 2006; Thum et al., 2007). We previously reported that treatment of T2DM stroke in male mice with exosomes harvested from human umbilical cord blood (HUCBC) derived CD133 + cells (early progenitor endothelial cells in cord blood, here referred to as CD133 + Exo), significantly improves cardiac function *via* reducing inflammation, oxidative stress, fibrosis, and increasing microRNA 126 expression at 28 days post-stroke compared to control T2DM-stroke mice (Venkat et al., 2021). Treatment of stroke in diabetic mice with miR-126 enriched EPC-exosomes administered intravenously at 2 h after stroke significantly reduces infarct size, improves neurological function, as well as increases cerebral blood flow and angiogenesis in the peri-infarct region compared to control T2DM-stroke mice (Wang et al., 2020). While the therapeutic effects of CD133 + Exo, EC-Exo, and EPC-Exo on stroke outcome and cardiac function have been previously reported, the effect of CD133 + Exo on the liver function in diabetic stroke mice has not been studied. In this study, we report for the first time that among the major peripheral organs, i.e., heart, kidney and liver, stroke elicits earlier, and stronger acute phase response in the liver of diabetic mice. We also present a novel therapeutic approach using CD133 + Exo not only to improve neurological and cognitive outcome post-stroke in T2DM mice but also to reduce the progression of NAFLD/NASH after stroke.

## Materials and methods

All procedures were carried out in accordance with the National Institutes of Health (NIH) Guide for the Care and Use of Laboratory Animals and with the approval of Institutional Animal Care and Use Committee (IACUC) of the Henry Ford Health System. This manuscript is prepared following ARRIVE guidelines (Kilkenny et al., 2010).

### Photothrombotic ischemic stroke model

The photothrombotic stroke model is a minimally invasive small vessel occlusion model which induces reproducible and well-defined infarcts in the frontal and parietal cortex and prolonged sensorimotor impairment in mice (Labat-gest and Tomasi, 2013; Chen et al., 2017; Uzdensky, 2018). Briefly, mice were anesthetized in a chamber with 3.5% isoflurane and maintained with 1.5% isoflurane in a mixture of 70% N_2_O and 30% O_2_ using a facemask. The head was fixed on the stereotaxic instrument and animals placed on a water circulating heating pad set to 37°C during the surgical procedure. A skin incision was made, and a black roundabout rubber sheet was placed on the skull surface such that the sensorimotor area (1.5–3 mm lateral; 0.5–1 mm anterior of bregma) was exposed by the inner circle aperture. A photosensitive dye (Rose Bengal, 40 mg/kg, i.p.) mixed with saline (4 ml/kg) was injected and 5 min later, a cold light illuminator was fixed close to the skull surface and turned on for 20 min. Activation of the dye induces endothelial damage with platelet activation and thrombosis, resulting in local blood flow interruption and infarction (Labat-gest and Tomasi, 2013; Uzdensky, 2018). The rubber was removed, incision was sutured, and routine post-surgical monitoring, analgesics (Buprenorphine SR, 1 mg/Kg, subcutaneous), and supportive care was provided.

### Experimental groups, randomization, and blinding

Diabetic women have a higher risk of stroke than men (Peters et al., 2014). The incidence of NAFLD is higher in males than in females as evidenced by longitudinal studies (Kojima et al., 2003; Ballestri et al., 2017). However, with advancing age (≥50 years), women have a higher risk of NASH and advanced NAFLD fibrosis than men (Summart et al., 2017; Balakrishnan, 2021). Thus, we employ female T2DM and control mice in this study. Female, adult, 3–4 m old, T2DM (BKS.Cg-m +/+ Leprdb/J, Jackson Laboratory, Bar Harbor, ME, USA) and control non-DM (db +, Jackson Laboratory, Bar Harbor, ME, USA) mice were used and following experimental groups employed: (1) Non-DM (WT, *n* = 6), (2) Non-DM-stroke (WT-Stroke, *n* = 15), (3) T2DM (*n* = 8), (4) T2DM-stroke (*n* = 12), and (5) T2DM-stroke + Exo (*n* = 13). CD133 + Exo (20 μg/200 μl PBS) was administered *via* a tail vein once at 3 days after stroke. The treatment regimen was selected based on our prior study in which we demonstrated that that treatment of T2DM stroke with CD133 + Exo administered once intravenously at 3 days after stroke at a dose of 20 μg/200 μl PBS significantly improves cardiac function at 28 days post-stroke compared to control T2DM-stroke mice (Venkat et al., 2021). At day 28 after stroke, fasting blood glucose was tested using a glucose analyzer (AgaMatrix Advanced blood glucose monitoring system) and blood lipids measured using CardioChek Plus analyzer. End-point measurements such as neurological and cognitive testing, histochemical staining, imaging, and quantification analysis were performed by investigators who were blinded to the experimental groups. The investigator who performed behavioral testing was not involved in performing stroke surgery or treatment administration.

### Neurological and cognitive function assessment

To assess neurological function post-stroke, the modified neurological severity score (mNSS) and adhesive removal tests were performed on days 1, 7, 14, 21, and 28 after stroke. The mNSS test is a composite of motor, sensory, balance, and reflex tests in which the absence of a tested reflex or abnormal response is scored as one point. Thus, on a scale of 0–18, 0 indicates normal neurological function, i.e., no deficits and a score of 18 indicates severe neurological deficits (Chen et al., 2001). The adhesive removal test evaluates somatosensory dysfunction (Bouet et al., 2009). Two small pieces of adhesive paper tapes were applied on both the forepaws with equal pressure and the time taken to sense the stimulus and to remove the tapes was recorded. The maximum trial time was 120 s. Three individual trials that were separated by at least 5 min were carried out for each animal on each testing day.

To evaluate cognitive impairment, novel object recognition (NOR) test and odor test were performed at 25–28 days after stroke using ANY-Maze video tracking and analysis software (Stoelting Co., Wood Dale, IL, USA). All animals were habituated to the testing environment 1 day prior to testing. To test short-term memory, a NOR test with a retention delay of 4 h was employed following previously described methods (Culmone et al., 2022). Briefly, in the 5 min trial phase, mice freely explored 2 identical plastic objects that were placed equidistant from the walls of a testing chamber. During the 5 min test phase, one object from the trial phase was replaced with a novel object and the time spent exploring each object was recorded. If the total exploration time was less than 10 s, animals were excluded from analysis. Sniffing, pawing, or probing with whiskers within 1 cm of an object was considered as exploration. The discrimination index was calculated as the ratio of time spent exploring the novel object to the total exploration time. To test long-term memory, odor test was employed following previously described methods (Spinetta et al., 2008; Culmone et al., 2022). Briefly, two sets of novel odors (N1 and N2) were obtained by placing beads in the home cage of donor mice (same sex, different strains) for 1 week. All experimental animals were separated to single housing and four wooden beads^1^ were placed in each cage to habituate mice to presence of beads and to collect familiar odor beads (F). On the 2 days of testing, animals were familiarized with novel odor N1 during three 1 min trials. On the 3 days of testing, mice were subjected to a 1 min test phase during which two familiar odor beads (F), one N1 odor bead and one N2 odor bead were placed in the center of the cage. The time spent exploring each odor bead (F, N1, N2) was recorded and discrimination index was calculated as the ratio of time spent exploring the novel odor N2 to the total time spent exploring all beads. Mice that were inactive and failed to explore any of the beads were excluded from the analysis.

### CD133 + Exo isolation

**FIGURE 1:**
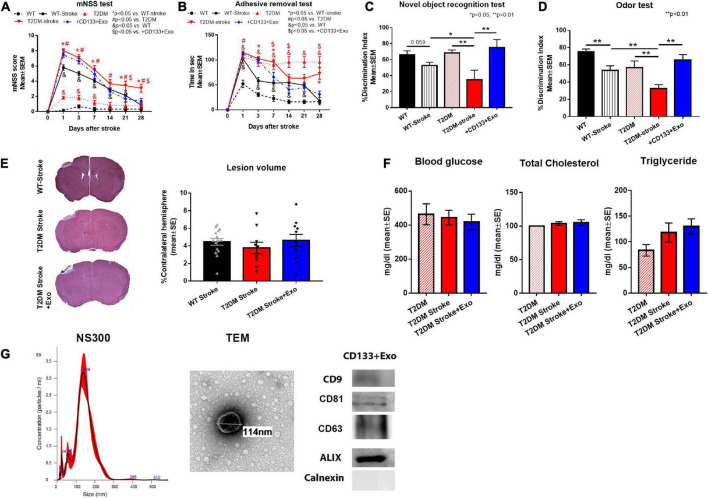
CD133 + Exo treatment significantly improves neuro-cognitive outcome in T2DM stroke mice. **(A,B)** Stroke in T2DM mice induced severe neurological deficits indicated by higher mNSS scores and longer adhesive removal times compared to T2DM sham and non-DM stroke (WT-stroke) mice. Intravenous administration of CD133 + Exo at 3 days after stroke reduced neurological impairment indicated by significantly lower mNSS scores on days 21 and 28 after stroke as well as significantly shorter adhesive removal time on days 7, 14, and 28 after stroke when compared to T2DM-stroke mice. **(C,D)** Stroke in T2DM mice induced significant cognitive impairment indicated by lower discrimination index in NOR test and odor test at 28 days after stroke compared to T2DM sham and WT-stroke mice. Treatment of stroke with CD133 + Exo significantly improved both short-term and long-term memory evaluated by NOR and Odor test compared to T2DM stroke mice. **(E)** There were no significant differences in the lesion volume among non-DM stroke, T2DM-stroke and CD133 + Exo treated T2DM-stroke mice. **(F)** There were no significant differences (*p* > 0.05) in blood glucose, total cholesterol or triglyceride levels at 28 days after stroke among T2DM, T2DM-stroke and CD133 + Exo treated T2DM-stroke mice. **(G)** Characterization of CD133 + Exo by NTA, TEM and Western blots. Non-DM (WT, *n* = 6), non-DM-stroke (WT-Stroke, *n* = 15), T2DM (*n* = 8), T2DM-stroke (*n* = 12), and T2DM-stroke + Exo (*n* = 13).

CD133 + cells (SER-CD34-F, ZenBio, Durham, NC, USA) were cultured using custom media (ZenBio, Durham, NC, USA) as per manufacturer instructions. Briefly, Stem Cell Expansion Supplement (100×) was thawed at room temperature and added to the Custom Stem Cell Expansion Medium at 1:100 ratio. CD133 + cells were cultured for 1 week and media was collected and filtered with 0.22 μM syringe filter to remove any particulate matter. Exosomes were harvested from collected media using ExoQuick (System Biosciences, Palo Alto, CA, USA). 2 ml ExoQuick was added for every 10 ml media and stored overnight at 4°C. Media was centrifuged at 1,500 *g* for 30 min, supernatant removed, and the pellet resuspended in PBS. Protein concentration was determined using BCA Protein Assay Kit (Pierce) (Thermo Fisher Scientific, Waltham, MA, USA) following standard protocol. CD133 + Exo were verified by transmission electron microscopy (TEM), western blot detection of common exosome marker proteins [CD9 (1:1000 Abcam, Waltham, MA, USA, Cat#223052), CD81 (1:1000 Abcam, Waltham, MA, USA, Cat#109201), CD63 (1:1000, Abcam, Waltham, MA, USA, cat# ab134045), ALIX (1:1000 Cell Signaling, Danvers, MA, USA, Cat#2171), and Calnexin (1:1000 Biolegend, San Diego, CA, USA, Cat#699401)] and exosome size and quantification were performed using NanoSight NS300 (Malvern Panalytical, Malvern, UK). Ultrastructure and nanosize analysis demonstrated that CD133 + Exo had intact spherical/donut-shaped morphology with a mean diameter of 144.3 ± 4.6 nm (Figure 1G).

### Immunohistochemistry and quantification analysis

Animals were anesthetized with ketamine (80 mg/kg, i.p) and (xylazine 13 mg/kg, i.p), transcardially perfused with 0.9% saline and decapitated. The brain was excised, immersion fixed in 4% paraformaldehyde overnight, cut into seven equally spaced (1 mm each) coronal blocks using a mouse brain matrix and embedded in paraffin. A series of adjacent 6 μm thick sections was cut from each block and stained with hematoxylin and eosin (H&E) for ischemic lesion volume measurement. Lesion volume was measured using ImageJ (NIH) and is presented as a volume percentage of the lesion area compared with the contralateral hemisphere. Brain coronal tissue sections (6 μm) were prepared and antibody against VWF (1:400; Dako) (Agilent Technologies, Santa Clara, CA, USA) and αSMA (α-smooth muscle actin; smooth muscle cell marker, mouse monoclonal IgG 1:800, Dako) were used (Agilent Technologies, Santa Clara, CA, USA). Bielschowsky silver and Luxol fast blue staining was used to stain axons and myelin, respectively. Control experiments consisted of similar procedures without addition of primary antibody. For each brain section, 6–8 fields of view of the ischemic border zone (IBZ) or ipsilateral striatum were digitized under a 20 × objective (Olympus BX40) using a 3-CCD color video camera (Sony DXC-970MD) interfaced with an MCID image analysis system (Imaging Research, St. Catharines, ON, Canada). Data were averaged to obtain a single value for each animal and presented as percentage of positive area or number of positive cells/mm^2^.

The left lateral lobe of the liver was excised and cut into three sections. One section was snap frozen in liquid nitrogen, one section was immersion fixed in 4% paraformaldehyde overnight and embedded in paraffin, and the third section was embedded in cryoprotective optimal cutting temperature compound (OCT) solution and flash frozen in 2-methyl butane on dry ice and then stored at −80°C. Oil red O staining of 15 μm thick frozen liver sections was used to evaluate neutral triglycerides and lipid content. H&E staining of 6 μm thick paraffin embedded sections was used to evaluate steatohepatitis and NAFLD Activity Score (NAS) which is the unweighted sum of scored for steatosis (0–3), lobular inflammation (0–3), and hepatocellular ballooning (0–2) (Kleiner et al., 2005; Trovato et al., 2014). Picrosirius red staining of 6 μm thick paraffin embedded sections was used to evaluate liver fibrosis.

### Alanine aminotransferase activity

During euthanasia, blood was collected under deep anesthesia *via* retro-orbital bleeding. Blood was allowed to clot at room temperature for 30 min and centrifuged at 1,500 *g* for 10 min to collect serum. Serum ALT activity was measured using an ALT Activity Assay kit (MAK052, MilliporeSigma, St. Louis, MO, USA) following manufactures instructions.

### Western blot

To test the acute phase response of peripheral organs after stroke, additional sets of T2DM-sham and T2DM-stroke mice were sacrificed at 1 and 3 days after stroke (*n* = 3/group). Animals were anesthetized with ketamine (80 mg/kg, i.p) and (xylazine 13 mg/kg, i.p), transcardially perfused with 0.9% saline and decapitated. The heart, liver and kidney were excised and snap frozen in liquid nitrogen. Protein was isolated from heart, liver and kidney tissue using Trizol (Thermo Fisher Scientific, Waltham, MA, USA) following standard protocol. Protein concentration was measured using the BCA kit (Thermo Fisher Scientific, Waltham, MA, USA) and 40 μg of protein/lane was loaded in a 10% SDS PAGE precast gel (Thermo Fisher Scientific, Waltham, MA, USA). The gel was placed in a tank and run for approximately 90 min at 120 V. The gel was subsequently transferred to a nitrocellulose membrane using the iBlot transfer system (Thermo Fisher Scientific, Waltham, MA, USA). The membrane was blocked in 2% I-Block (Thermo Fisher Scientific, Waltham, MA, USA) in 1 × TBS-T for 1 h, and then primary antibodies against either β-actin (1:10,000, Abcam, Waltham, MA, USA, cat# ab6276), serum amyloid A (SAA, 1:1000, Abcam, Waltham, MA, USA, cat # ab199030), NOD-like receptor protein 3 (NLRP3, 1:1000, Cell Signaling, Danvers, MA, USA, cat# 15101), Interleukin-1β (IL-1β, 1:1000, Abcam, Waltham, MA, USA, cat# ab2105), IL-6 (1:1000, Fisher Scientific, Hampton, NH, USA, cat# 700480), Tumor necrosis factor α (TNFα, 1:1000, Abbiotec, Escondido, CA, USA, cat# 250844), Toll like receptor 4 (TLR4, 1:500, Santa Cruz, Dallas, TX, USA, cat# sc-10741) were used. Secondary antibodies (anti-mouse, Jackson ImmunoResearch, West Grove, PA, USA) were added at 1:3,000 dilution in 2% I-Block in 1 × TBS-T at room temperature for 1 h. The membranes were washed with 1 × TBS-T, and then Luminol Reagent (Santa Cruz, Dallas, TX, USA) was added. The membranes were then developed using a FluorChem E Imager system (ProteinSimple, San Jose, CA, USA). Grayscale images were analyzed using ImageJ and normalized to β-actin.

### Statistical analysis

Data are presented as mean ± SEM. Data were evaluated for normality; ranked data were used for analysis when data were not normally distributed. To study the combination of T2DM and stroke effects, we used two factorial design with factors T2DM and stroke and Analysis of variance (ANOVA) was used for single measurements collected at day 28 (NOR test, odor test, and immunostaining measurements) and analysis of variance and covariance (ANCOVA) was used for repeated measurements (mNSS and adhesive removal test). The analysis would first test two-factor interaction, followed by assessment of additive effect (no interaction), supper-additive or sub-additive interaction effects. Repeated analysis of variance (ANCOVA) was used to test CD133 + Exo effect on function recovery for mNSS and adhesive removal test in mice with both T2DM and stroke. Statistical significance was detected at *p* < 0.05. Data analysis was performed by a Biostatistician.

## Results

### CD133 + Exo treatment significantly improves neuro-cognitive outcome in T2DM stroke mice

Stroke in T2DM mice induced severe neurological deficits (Figures 1A, B) indicated by higher mNSS scores and longer adhesive removal times as well as significant cognitive impairment (Figures 1C, D) indicated by lower discrimination index in NOR test and odor test compared to T2DM sham and non-DM stroke mice. Super-additive effect was observed on mNSS scores at day 28 and sub-additive effects were observed on the adhesive removal test on days 1, 7, 14, and 21 after stroke. Treatment of stroke with CD133 + Exo significantly reduced neurological impairment and improved both short-term and long-term memory compared to T2DM stroke mice (Figures 1A-D). However, there were no significant differences (*p* > 0.05) in the lesion volume among non-DM stroke, T2DM-stroke and CD133 + Exo treated T2DM-stroke mice (Figure 1E). There were no significant differences (*p* > 0.05) in blood glucose, total cholesterol or triglyceride levels at 28 days after stroke among T2DM, T2DM-stroke and CD133 + Exo treated T2DM-stroke mice (Figure 1F).

### CD133 + Exo treatment of T2DM stroke significantly increases vascular and white matter/axon remodeling

**FIGURE 2:**
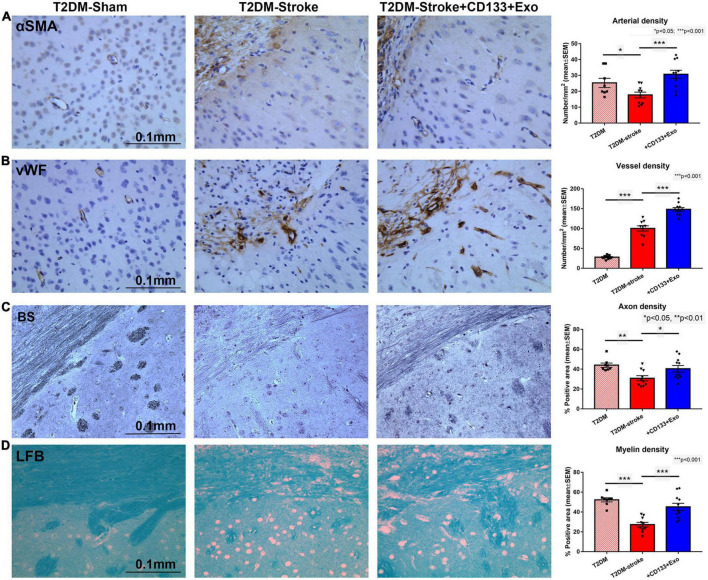
CD133 + Exo treatment of T2DM stroke significantly increases vascular and white matter/axon remodeling. **(A,B)** CD133 + Exo treatment of T2DM-stroke significantly increases arterial blood vessel density (αSMA) and vessel density (vWF) in the ischemic brain compared to T2DM-stroke mice. **(C,D)** CD133 + Exo treatment of T2DM-stroke significantly increases axon density (Bielschowsky silver, BS) and myelin density (Luxol fast blue, LFB) in the ischemic brain compared to T2DM-stroke mice at 28 days after stroke. T2DM (*n* = 8), T2DM-stroke (*n* = 12), and T2DM-stroke + Exo (*n* = 13).

To test the effects of CD133 + Exo treatment on vascular and white matter/axonal remodeling in the ischemic brain, we measured arterial blood vessel and vascular density in the IBZ based on αSMA and vWF immunostaining, respectively, and myelin and axon density in the ipsilateral white matter striatal bundles using Luxol fast blue and Bielschowsky silver staining, respectively. T2DM-stroke significantly alters vascular density and reduces axon and myelin density in the IBZ compared to T2DM Sham. CD133 + Exo treatment of T2DM-stroke significantly increased arterial blood vessel density, vessel density, myelin density and axon density in the ischemic brain compared to T2DM-stroke mice at 28 days after stroke (Figures 2A-D).

### T2DM-stroke induces liver immune-response earlier and stronger than in other peripheral organs

**FIGURE 3:**
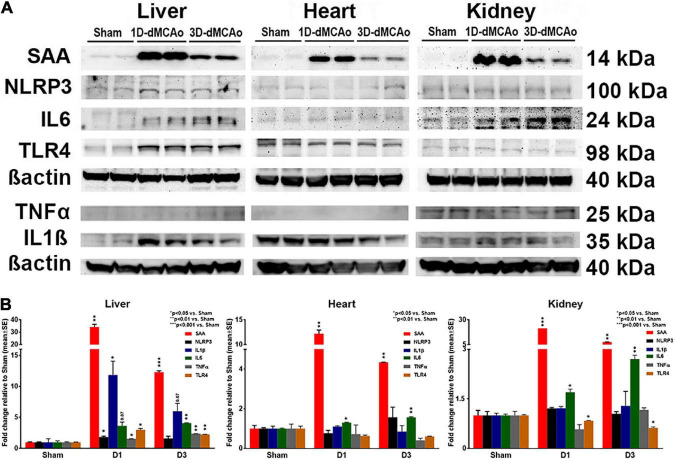
T2DM-stroke induces liver immune-response earlier and stronger than in other peripheral organs. **(A,B)** Western blot was used to measure the expression of inflammatory factors, cytokines, and acute phase response related proteins at 1 and 3 days after stroke in T2DM mice. Stroke in T2DM mice induces earlier and higher cytokine and acute phase response protein expression such as SAA, NLRP3, IL1β, IL6, TNFα and TLR4 in the liver compared to heart and kidney. The bands correspond to the precursor form for IL1β and TNFα. *n* = 3/group.

The acute phase response is typically initiated in response to most inflammatory triggers to coordinate the innate immune response (McColl et al., 2007). Triggers for the acute phase response include elevations of cytokines IL-1β, IL-6, and TNFα, which can bind directly to hepatocytes and induce the synthesis and subsequent release of acute phase response proteins (McColl et al., 2007). Therefore, we examined the expression of inflammatory factors, cytokines and acute phase response related proteins at 1 and 3 days after stroke in T2DM mice. Figure 3 data show that stroke in T2DM mice induced earlier and higher inflammatory factor, cytokine, and acute phase response protein expression such as SAA, NLRP3, IL1β, IL6, TNFα and TLR4 in the liver compared to heart and kidney, as measured by Western blot. Combination of T2DM and stroke had a super-additive effect on the increased expression of SAA and IL6.

### Stroke in T2DM-mice exacerbates NAFLD progression while CD133 + Exo treatment significantly reduces steatosis, fibrosis, NAS, and ALT activity

**FIGURE 4:**
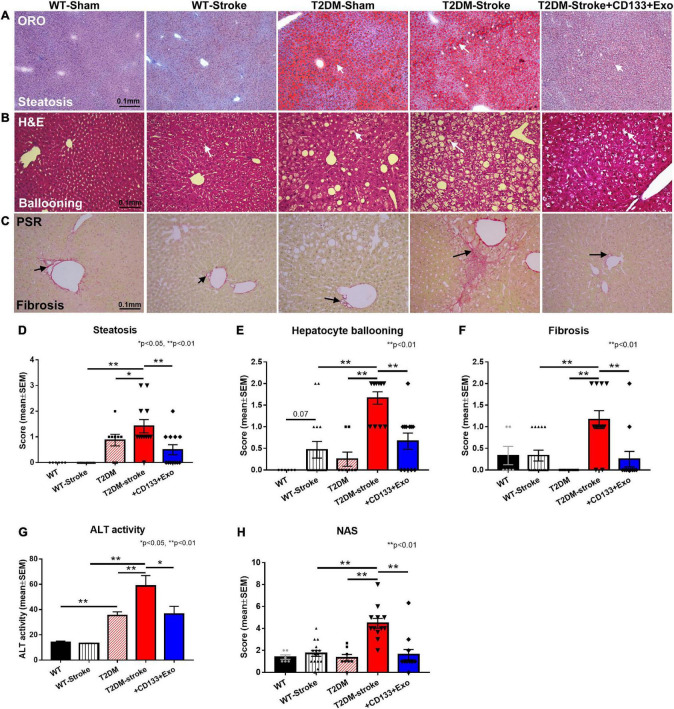
Stroke in T2DM-mice exacerbates NAFLD progression while CD133 + Exo treatment significantly reduces steatosis, fibrosis, NAS, and ALT activity. **(A–C)** Representative images of Oil red O (ORO), hematoxylin and eosin (H&E) staining and PicroSirius Red (PSR). T2DM-stroke mice exhibit significantly increased liver steatosis **(D)**, increased ballooning degeneration of hepatocytes **(E)**, increased fibrosis **(F)**, increased serum ALT activity **(G)**, and higher NAS **(H)** compared to T2DM-Sham mice. CD133 + Exo treatment of T2DM-stroke mice significantly decreased liver steatosis, hepatocellular ballooning, fibrosis, serum ALT activity, and NAS compared to T2DM-stroke mice at 28 days after stroke. NAS is the unweighted sum of scores for steatosis (0–3), lobular inflammation (0–3) and hepatocellular ballooning (0–2). Non-DM (WT, *n* = 6), non-DM-stroke (WT-Stroke, *n* = 15), T2DM (*n* = 8), T2DM-stroke (*n* = 12), and T2DM-stroke + Exo (*n* = 13).

Since NAS is a composite of steatosis, lobular inflammation, and hepatocellular ballooning (Kleiner et al., 2005; Trovato et al., 2014), higher NAS scores indicate more severe NAFLD. Figure 4 shows that T2DM-stroke mice exhibited significantly increased liver steatosis measured by Oil red O staining, increased ballooning degeneration of hepatocytes, increased fibrosis measured by PicroSirius Red, increased serum ALT activity, and higher NAS compared to T2DM-Sham mice, with super-additive effect observed on hepatocyte ballooning, fibrosis, NAS and ALT. CD133 + Exo treatment of T2DM-stroke mice significantly decreased liver steatosis, hepatocellular ballooning, fibrosis, serum ALT activity, and NAS compared to T2DM-stroke mice at 28 days after stroke (Figures 4A-H). Thus, CD133 + Exo treatment significantly attenuates the progression of NAFLD/NASH in T2DM stroke mice.

## Discussion

In this study, we report for the first time that stroke in T2DM mice induces acute phase immune response earlier and stronger in the liver compared to heart and kidney and worsens NAFLD compared to T2DM sham or stroke in non-DM mice. We also report that treatment of T2DM stroke with CD133 + Exo not only improves neuro-cognitive outcome but also attenuates the progression of NAFLD/NASH, thereby holding promise as a novel therapeutic agent for the treatment of T2DM stroke, and potentially, directly for NAFLD/NASH.

There is increasing evidence of the clinical importance of dysfunctional brain–peripheral organ interactions (Anthony et al., 2012; Ren et al., 2018; Zhao et al., 2019; Li et al., 2020; Yan et al., 2020). Our prior work, as well as others have demonstrated that stroke in non-DM and T2DM mice induces significant and progressive cardiac dysfunction compared to corresponding control mice (Yan et al., 2020; Chen et al., 2021). T2DM aggravates such cerebral-cardiac syndrome (Lin et al., 2021; Venkat et al., 2021). Acute ischemic stroke patients who developed acute kidney injury had higher risk of mortality at 3 months after stroke (Qureshi et al., 2020; Zhao et al., 2020). Thus, we tested the expression of acute phase proteins and inflammatory factors and cytokines in kidney, heart and liver tissue at 1 and 3 days after stroke. Acute phase proteins are a class of proteins whose concentration dramatically increases, or increases in the circulation in response to stimulation by pro-inflammatory cytokines (Güleç et al., 2022). IL-6 is the major inducer for hepatic secretion of acute phase proteins while IL-1β, TNFα and interferon gamma (IFN-γ) are other cytokines that mediate acute phase response (Güleç et al., 2022). In this study, we report for the first time that stroke in T2DM mice induces earlier and higher inflammatory factor, cytokine, and acute phase response protein expression such as SAA, NLRP3, IL-1β, IL-6, TNFα, and TLR4 in the liver compared to heart and kidney. This novel finding improves our understanding of the brain-peripheral organ interaction sequelae and may impact the treatment of diabetic stroke. The liver is the principal organ contributor to circulating level of chemokines and acute phase protein, SAA after focal brain injury (Wicker et al., 2019). After acute brain injury, chemokine expression by the liver results in neutrophil recruitment and hepatic damage (Campbell et al., 2005, 2008; Nizamutdinov et al., 2017), contributing to multi-organ dysfunction (Villapol, 2016), and amplification of the local injury responses (Campbell et al., 2008; Villapol, 2016). SAA is synthesized in the liver and is induced in response to pro-inflammatory stimuli such as IL-1β, IL-6, and TNFα. Serum SAA increases more than 1,000 fold during severe acute-phase inflammation, and SAA is modestly elevated in chronic inflammation (Shridas et al., 2018). SAA has pro-inflammatory activities and participates in systemic modulation of innate and adaptive immune responses, lipid metabolism/transport, metabolic endotoxemia, obesity and insulin resistance, and in the induction of extracellular-matrix-degrading enzymes (Li et al., 2016; Griffiths et al., 2017). Thus, SAA has the potential to serve as a novel, early plasma biomarker for T2DM and NAFLD (Li et al., 2016; Griffiths et al., 2017). SAA has strong chemotactic activity for neutrophils and macrophages, induces the expression of a variety of inflammatory cytokines, and stimulates NLRP3 inflammasome activity, mediated in part by TLR4 (Chami et al., 2019; Yu et al., 2019). Therefore, early inflammation in the liver is likely a key contributor to post-stroke NAFLD progression in T2DM mice.

Stroke in diabetic patients elicits a strong bilateral brain-liver interaction, with significant associations between changes in biomarkers of liver dysfunction, ischemic lesion volume and systemic inflammation (Muscari et al., 2014; Cassidy et al., 2015; Abdeldyem et al., 2017; Li et al., 2017, 2018; Weinstein G. et al., 2018; Weinstein et al., 2019). Immune communication between the brain and peripheral organs contributes to and amplifies the pathophysiological sequelae of stroke and neural injury (Maier et al., 2006; Lu et al., 2009; Denes et al., 2010; Anthony et al., 2012; Tobin et al., 2014; Wendeln et al., 2018). The liver is a key organ mediating the inflammatory interaction between brain and peripheral organs (Decker et al., 1985; Freitas-Lopes et al., 2017). Preclinical and clinical data indicate that brain injury induces liver damage and hepatic inflammation (Villapol, 2016; Zhang et al., 2018). Acute stroke increases liver damage identified by increased serum ALT at 3 h after middle cerebral artery occlusion stroke in rats (Yang et al., 2003), and hyperlipidemia exacerbates liver damage by promoting oxidative stress, inflammation and hepatocyte apoptosis (Gong et al., 2012). Focal brain injury elicits a rapid hepatic response and the subsequent production of chemokines by the liver acts as an amplifier of the focal cerebral injury (Anthony et al., 2012; Villapol, 2016; Zhang et al., 2018). Post-stroke inflammatory response in the brain changes over time, and the elevation of proinflammatory factors and cytokines in the CNS can precede or follow elevated levels in the blood (Maier et al., 2006; Lu et al., 2009; Anthony et al., 2012; Tobin et al., 2014; Shi et al., 2021). NAFLD when accompanied by fibrosis is strongly associated with systemic inflammation and elevation of hepatic as well as plasma IL-6 expression (Wieckowska et al., 2008). Liver fibrosis is associated with a higher risk of ischemic stroke particularly in women (Bateman et al., 2016). Our data indicate that female T2DM mice subjected to stroke exhibit significantly increased serum ALT activity and increased hepatic fibrosis, steatosis and NAFLD/NASH progression compared to T2DM control and non-DM stroke mice. In addition, our data show that treatment of T2DM stroke with CD133 + Exo significantly improves neurological function, cognitive function, white matter and vascular remodeling in the ischemic brain as well as reduces hepatic injury and progression of NAFLD/NASH compared to T2DM stroke mice.

### Limitations and future studies

The role of the gut microbiota in mediating stroke pathogenesis and outcome is emerging. However, in this study, we only tested acute phase response in the heart, kidney and liver and found that among these major organs, stroke elicits stronger and earlier acute phase response in the liver. Given the increase in post-stroke morbidity, mortality, and neurological and cognitive deficits in the diabetic population and the global prevalence of NAFLD, it is important to investigate how cerebral ischemic stroke exacerbates NAFLD in T2DM. In a retrospective, hospital-based observational study of ischemic stroke patients, 64.2% (206/321) patients presented with NAFLD (Baik et al., 2019). These stroke patients with NAFLD had less severe strokes with lower NIHSS scores and favorable functional outcome at 90 days after stroke (Baik et al., 2019). However, as the study authors point out, the proportion of patients with comorbidities such as diabetes, atrial fibrillation, or peripheral arterial occlusive disease, was similar or even lower in the group of NAFLD-stroke patients compared to stroke patients without NAFLD (Baik et al., 2019). Therefore, further studies are needed to verify the effect of NAFLD on stroke outcomes in diabetic population as well as evaluate whether NAFLD is aggravated post-stroke and its effect on stroke outcome. The mechanisms by which CD133 + Exo treatment improves neurocognitive recovery as well as reduces NAFLD/NASH progression, and how liver dysfunction induced by T2DM stroke possibly feeds-back to amplify neurological dysfunction have not been investigated in the current study. NAFLD is characterized by triglyceride accumulation in the cytoplasm of hepatocytes and measurement of hepatic triglycerides and free fatty acids as well as evaluation of the therapeutic effects of CD133 + Exo in a T2DM only model and NASH only model is warranted. In addition, studies investigating sex and age dependence of T2DM stroke on liver dysfunction and therapeutic response to CD133 + Exo treatment warrant investigation.

## Conclusion

Stroke in T2DM mice induces acute phase immune response earlier and more intensely in the liver compared to heart and kidney. Stroke in T2DM mice induces significant NAFLD indicated by increased steatosis, hepatocellular ballooning, inflammation, and fibrosis in the liver. CD133 + Exo treatment significantly improves neurocognitive function, vascular and white matter remodeling in the ischemic brain as well as attenuates the progression of NAFLD/NASH in the liver of T2DM stroke mice. Thus, CD133 + Exo is a novel therapeutic agent that can improve both brain and liver function after stroke in T2DM mice.

## Disclosures

Intellectual Property relating to the topic of this manuscript is subject to patent application (62/586,102) fully owned by Henry Ford Health System.

## Data availability statement

The original contributions presented in this study are included in the article/supplementary material, further inquiries can be directed to the corresponding author.

## Ethics statement

This animal study was reviewed and approved by the Institutional Animal Care and Use Committee (IACUC) of Henry Ford Health.

## Author contributions

PV: writing—original draft preparation, visualization, and formal analysis. HG, EF, AZ, and JL-W: investigation. ZC: investigation and formal analysis. BP: behavioral testing. ZZ: writing—review and editing and supervision. ZL: investigation and writing—review and editing. ML: formal analysis (statistical). MC: conceptualization, writing—review and editing, and supervision. All authors read and agreed to the published version of the manuscript.

## Footnotes

1
https://www.craftparts.com/
